# Decease of Prof. Patterson and Dr. J. Kearney Rodgers

**Published:** 1851-12

**Authors:** 


*Decease of Prof. Patterson and Dr. J. Kearney Rodgers.*— These two distinguished members of the profession, have recently paid the debt of nature. Prof. Patterson was born and educated in Scotland, and came to this country about twenty years ago. He was formerly connected with the Jefferson Medical College, but for several years has been Professor of Anatomy in the University of the city of New York. He has long enjoyed a high reputation as a teacher of anatomy, and his demise will be felt as a great loss to the institution with which he was connected at the time of his death. He died after a short illness, of the nature of which we have not been informed.

The late Dr. Rodgers, has for a long period occupied a prominent position as a practicing surgeon in the city of New York. He has for many years officiated as one of the Surgeons of the New York Hospital. He sustained an exalted characted as a high minded, honorable man, and in all the relations of life his example was worthy of imitation. The editor of the N. Y. Med. Gazette remarks that " he died as he lived, a christian man, and his bereaved family and friends have the consolation of believing that our loss is his gain."

We are informed that the fatal disease was *portal phlebitis*, a very rare a ffection, but few cases being on record.

---

*New Medical College.*— A new medical college has been organized at Cincinnati, Ohio, and a lecture session is announced for the present autumn.

